# The Role of Rumination and Stressful Life Events in the Relationship between the Qi Stagnation Constitution and Depression in Women: A Moderated Mediation Model

**DOI:** 10.1155/2017/7605893

**Published:** 2017-07-03

**Authors:** Mingfan Liu, Ying Jiang, Xiumei Wang, Qiaosheng Liu, Hou Wu

**Affiliations:** ^1^Center of Mental Health Education and Research, Jiangxi Normal University, Nanchang 330022, China; ^2^Department of Psychology, Jiangxi Normal University, Nanchang 330022, China; ^3^Clinical Psychological Center, Psychiatric Hospital of Jiangxi Province, Nanchang 330000, China; ^4^Nanchang Institute of Technology, Nanchang 330000, China

## Abstract

The qi stagnation constitution is associated with depression in traditional Chinese medicine. It is unclear how rumination and stressful life events affect the relationship between the qi stagnation constitution and depression. The Qi Stagnation Constitution Scale, Ruminative Response Scale, Center for Epidemiologic Studies Depression Scale, and Adolescent Self-Rating Life Events Checklist were used to assess this association in 1200 female college students. The results revealed that the qi stagnation constitution was positively associated with depression. Furthermore, rumination was a partial mediator of the relationship between the qi stagnation constitution and depression. In addition, stressful life events moderated the direct effect and mediating effect of the qi stagnation constitution on depression. These findings indicate that rumination and stressful life events may affect the relationship between the qi stagnation constitution and depression in women.

## 1. Introduction

Depression accounts for the largest share of the world's burden of disease as measured by years lost to disability [1]. It is even more common among women than among men [2, 3]. Approximately twice as many women as men meet the criteria for major depressive disorder [2, 4]. The fact that women are more prone to depression than men across many nations and cultures suggests that biological factors account for the gender difference in depression [2]. According to theories of traditional Chinese medicine, biological explanations usually focus on the effect of body constitution [5, 6]. Constitution, a physiological component that is formed based on congenital and acquired factors, has been found to affect individual susceptibility to a specific disease [7–9]. Researchers found that some constitutions, including qi stagnation, qi deficiency, and yang deficiency, had a high tendency to be associated with depression [10, 11]. Among them, the qi stagnation constitution was considered to greatly increase the risk of depression [12, 13]. The common symptoms of a body with the qi stagnation constitution are as follows: often melancholy and being easily stressed, frequent sighing, swollen breasts, heart palpitations, the feeling that something is stuck in the throat, and susceptibility to insomnia and depression [14]. Researchers have suggested that women have a higher rate of the qi stagnation constitution than men [15, 16]. An epidemiological investigation of constitution found a prevalence of qi stagnation of 11.5% in women and 7.1% in men [15]. In traditional Chinese medicine, qi (vital energy), which is believed to vitalize, propel, and warm the body, depends on the regulation of the liver [17, 18]. In addition, the liver balances emotions [17, 19]. If the liver conducts and performs functions abnormally, qi is impeded and induces emotional abnormalities such as depression [18]. Individuals, especially women who have a higher rate of qi stagnation than men, are more prone to depression because their livers lose the ability to function smoothly [18]. Therefore, the qi stagnation constitution is a powerful risk factor for depressive disorder among women. Several empirical studies have confirmed the strong link between qi stagnation constitution and depression [10–12]. Those studies investigated the bivariate relationships between these concepts only. However, relationships among variables are often more complex than simple associations [20]. To the best of our knowledge, no empirical studies have been conducted to identify psychological processes that link qi stagnation constitution to depression.

Rumination, as a general mediator of the relationship between many vulnerability factors and depression [21, 22], may be a plausible candidate for a mediator of the association between qi stagnation constitution and depression. Rumination is not only characterized as a response style that focuses one's attention on the causes and consequences of one's emotional state without attempting to engage in problem solving or active coping [23], but regarded as one of seven emotions (joy, anger, anxiety, pensiveness, grief, fear, and fright) in traditional Chinese medicine [18]. Chinese medicine believes that the seven emotions develop on the foundation of the physiological functions of the zang viscera [18, 24]. If the zang viscera's function is balanced and the body constitution maintains sturdiness, then normal and optimistic mind can be achieved. Conversely, if the zang viscera's function is imbalanced and human body becomes weak, mental state would be abnormal [24]. Qi stagnation constitution, which is viewed as an increase in “visceral restlessness” [18], is easily prone to rumination. Indeed, there is no lack of similar descriptions of ruminations about qi stagnation constitution such as “ascetic meditation,” “endlessly thinking,” and “contemplation” in ancient Chinese medicine books [14]. In other words, qi stagnation constitution has an important influence on a ruminative response style. Subsequently, according to the response style theory of depression, individuals with a ruminative response style are far more likely to suffer from depression [23, 25]. As conceived by Nolen-Hoeksema and his colleagues, rumination worsens and prolongs depression by exacerbating negative mood directly, interfering with effective problem solving, inhibiting instrumental behavior, and losing social support [25]. The strong relationship between rumination and depression has been powerfully supported in numerous research studies (see for reviews [25, 26]). Taken together, we hypothesized that qi stagnation would be related to an increase in rumination, which, in turn, would be associated with depression. That is, rumination may mediate the relationship between qi stagnation and depression.

Nevertheless, not all people with the qi stagnation constitution succumb to depression in real life [11]. Diathesis-stress theories of depression predict that vulnerable individuals' sensitivity to depression depends on whether they encounter a stressful life episode, which activates the potential for predisposition [27]. A great deal of empirical evidence supports this prediction. The risk of depression after a stressful experience is elevated for people who have certain preexisting traits (diatheses) such as adverse biological and cognitive factors [28]. Stress, which could produce blocked emotions, is one of the most common causes of liver qi stagnation in Chinese medicine [18]. People with qi stagnation constitution that is characterized by adverse biological factor may be more sensitive to stress and easily enter into emotional frustration [14] when they encounter more stressful life events. Chinese medicine believes that, compared with people who do not experience a stressful life event, those who experience more stressful life events can cause the liver qi to stagnate more severely [18], which in turn worsens depression. The present study focuses on the qi stagnation constitution as a potential diathesis and tests whether stressful life events moderate the influence of the qi stagnation constitution on depression.

The Chinese medicine believes that the main cause of rumination for qi stagnation constitution is principally due to blockage of the liver qi, which leads to the spleen losing its transportation and transformation functions [18]. Spleen disharmony is related to rumination. In other words, rumination may be the natural and spontaneous response of qi stagnation constitution. Therefore, on account of considering that rumination is a stable psychological characteristic of qi stagnation constitution [14] and will not be easily affected by stressful life events, this moderating effect of stressful life events between qi stagnation constitution and rumination may be small or nonsignificant. However, investigators have reported that individuals who experience a high number of extreme life events were found to interact with a high level of rumination predicted severe depressive symptoms [29–31]. It means that stressful life events moderate the effect of rumination on depressive level. Moreover, the magnitude of this relationship was stronger for women than for men [31]. Therefore, beyond thinking about the direct moderating effect, the indirect moderating effect of life events on the relationship between qi stagnation constitution and depression in women should also be investigated.

**Figure 1:**
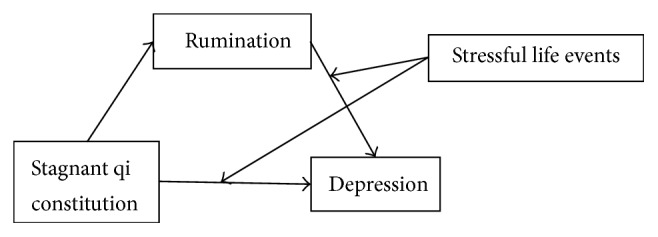
A moderated mediation model of qi stagnation constitution, rumination, life events, and depression.

This study aimed to examine the mechanism that explained the relationship between qi stagnation constitution and depression and conditional features such as “how,” “in what way,” and “under what circumstances.” Based on previous studies, a moderated mediation model was established (see Figure 1) to elucidate the relationship between the qi stagnation constitution and depression in female participants. Our hypotheses were as follows: (1) rumination would mediate the relationship between the qi stagnation constitution and depression in women. (2) Stressful life events would moderate the direct effect and indirect effect between the qi stagnation constitution and depression in women.

## 2. Method

### 2.1. Subjects

In this study, 1200 female subjects were recruited from two universities. Fifty-six students were excluded due to missing data for items on the scales. A total of 1144 participants ultimately completed all the surveys. The average age was 19.88 years (standard deviation (SD) = 1.66). Of the students, 44.3% were freshmen, 17% were sophomores, 32.7% were juniors, and 6% were at the master's level. In terms of residence, 28.2% of the students were living in the city, 37.5% were living in the county, and 34.3% were living in the countryside. All subjects provided written informed consent, and the study procedures were approved by the local ethics boards.

### 2.2. Instruments

#### 2.2.1. Measurement of Qi Stagnation Constitution

The Qi Stagnation Constitution Scale is a subscale of the constitution in Chinese Medicine Questionnaire developed by Wang et al. [32]. The subscale includes 7 items to assess individual constitutional differences based on characteristics such as bodily sensations and psychological characteristics (e.g., “fullness in the chest,” “abnormal sensation in the throat that seemed as though something was stuck,” and “feeling vulnerable or sentimental”) [33]. This subscale has been widely used to measure the qi stagnation constitution in China [34]. It was found to have good validity and reliability in Chinese [35, 36]. Cronbach's alpha coefficient in the present study was 0.74.

#### 2.2.2. Measurement of Rumination

The 22-item Ruminative Response Scale was used to measure the response style to negative mood [37]. Participants were required to report how often they felt this way on a four-point Likert-type scale. The higher the total score is, the more the individual engages in rumination. The Ruminative Response Scale was adapted to Chinese [38]. The reliability and validity of the adapted version have been well demonstrated in Chinese adolescent [38] and Chinese depressed patients [39]. Cronbach's alpha coefficient in this study was 0.87.

#### 2.2.3. Measurement of Stressful Life Events

The Adolescent Self-Rating Life Events Checklist (ASLEC) was developed by Liu and colleagues [40], who combined the characteristics of Chinese teenagers based on Chinese and foreign literature. It consists of 27 items, each of which typifies a stressful life event such as “interpersonal stress” and “suffering from punishment.” If participants have experienced an event, they are asked to rate the extent to which it influenced them over the last 12 months. Items are evaluated on a scale of 1 (did not influence me at all) to 5 (influenced me extremely). The reliability and validity of the ASLEC have been supported in samples of Chinese college students [41]. Cronbach's alpha coefficient was 0.90 in the current study.

#### 2.2.4. Measurement of Depressive Symptoms

The Center for Epidemiologic Studies Depression (CES-D) Scale [42] is a widely applied instrument of depressive symptoms occurring within the past week. The CES-D Scale was adapted to Chinese and demonstrated sound psychometric properties in different groups in China [43, 44]. It consists of 20 items on a 4-point scale to measure the frequency of occurrence. Cronbach's alpha coefficient in this study was 0.80.

### 2.3. Procedures

Participants were recruited from two universities. They completed paper measures during class time (approximately 40 min). Classrooms were visited by the same investigator to hand out the measures, explain the purpose of the study, and collect the measures. All participants were voluntary and their responses would be kept confidential. Finally, they were given course credit for their participation.

### 2.4. Statistical Analysis

All data were double-entered and cross-validated in EpiData Analyses which were conducted in SPSS 21. Descriptive statistics and correlations between study variables, including the dependent variable (depressive symptom), mediating variable (rumination), independent variable (qi stagnation constitution), and moderating variable (stressful life events), were calculated. Then, mediation model and moderated mediation model analyses were performed [45, 46]. Mediation manifests when an independent variable affects a mediator, which, in turn, affects the dependent variable [46]. A moderated mediation model refers to a mediation model that includes an additional moderator in one or more paths [47]. The present study was conducted using the PROCESS macro [45, 46]. Variables were mean centered prior to the analysis. To test the indirect effects and conditional indirect effects, a bootstrapping approach was employed (*n* = 5,000) [45, 46]. According to the recommendation of Hayes [45], unstandardized coefficients, which are the preferred metric in causal modelling, were reported in the present study. Additionally, because controlling for demographic variables did not change the pattern of the results, we reported models without demographic variables included.

## 3. Results

The descriptive statistics and Pearson correlation coefficients are presented in Table 1. The correlations revealed that depression was positively and significantly correlated with stressful life events, rumination, and the qi stagnation constitution (*r* = 0.31, *r* = 0.39, and *r* = 0.48, resp.; *p* < 0.01). These results indicated that women who experienced more stressful life events, more excessive rumination, or more obvious qi stagnation were more prone to depression. Multicollinearity did not appear due to appropriate variance inflation factors (less than 1.6).

To test the first hypothesis, as shown in Table 2, a mediational analysis was conducted. The positive effects of the qi stagnation constitution on rumination (*β* = 1.21, *t* = 23.20, *p* < 0.001) and of rumination on depression were significant (*β* = 0.14, *t* = 5.64, *p* < 0.001). The indirect effect of rumination on the relationship between the qi stagnation constitution and depression was significant (CI = [0.10, 0.25]). Despite the significant indirect effect, the direct effect between the qi stagnation constitution and depression remained significant (*β* = 0.66, *t* = 12.19, *p* < 0.001), which indicated that the mediation was partial.

Prior to testing for the second hypothesis, we examined whether the effect of qi stagnation constitution on female rumination was moderated by stressful life events. The analysis showed that the moderating effect of stressful life events on the relation of qi stagnation constitution and female rumination was nonsignificant (*β* = −0.002, *t* = −0.63, *p* = 0.53).

**Figure 2:**
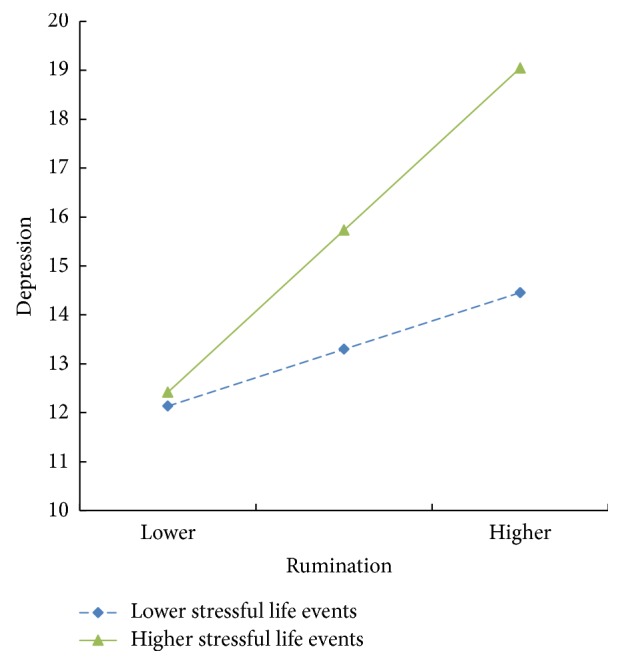
Interaction of rumination and stressful life events predicting depression.

**Figure 3:**
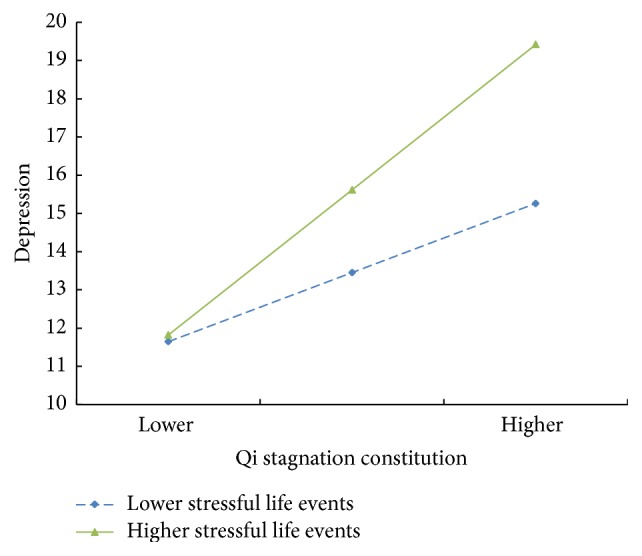
Interaction of qi stagnation constitution and stressful life events predicting depression.

To test the second hypothesis, as shown in Table 3, the interaction effects of rumination × stressful life events (*β* = 0.01, *t* = 3.04, *p* < 0.01) and the qi stagnation constitution × stressful life events (*β* = 0.01, *t* = 3.13, *p* < 0.01) on depression were significant. A simple slope test was also undertaken to plot the interaction effect at one standard deviation below and above the mean of the moderator. As shown in Figure 2, the relationship between rumination and depression is stronger when stressful life events are high (1 SD above the mean) and weaker when stressful life events are low (1 SD below the mean). Also, the relation between qi stagnation constitution and depressions is stronger when stressful life events are high and weaker when stressful life events are low (Figure 3).

Specifically, the bootstrapping procedure was employed to gain further insight into the direct and indirect effect of qi stagnation constitution on depression. As shown in Table 3, the conditional direct effect of the qi stagnant constitution on depression of highly and slightly stressful life events showed that although the qi stagnant constitution significantly predicted depression at both high and low stress levels, the magnitude of this relationship for high stress levels was markedly stronger (CI = [0.59, 0.85]) than for low stress levels (CI = [0.25, 0.56]). For the conditional indirect effect of the qi stagnant constitution on depression, a positive indirect effect was significant for highly stressful life events (CI = [0.12, 0.35]). However, the indirect effect was not significant for slightly stressful life events (CI = [−0.02, 0.14]). These results indicated that both the direct and indirect effects of the qi stagnation constitution on depression depended on stressful life events. The full model explained 32% of the variance in women's depression.

## 4. Discussion

The present study was the first to examine the role of rumination and stressful life events in the relationship between the qi stagnation constitution and depression in women using a moderated mediation model. This study specifically applied interdisciplinary perspectives to combine the theory of Chinese medicine with clinical psychology, which can extend the findings of depression research and provide empirical support for the theory of traditional Chinese medicine. The results confirmed that the qi stagnation constitution is a significant risk factor for depression. Furthermore, the available evidence of mediation (rumination) and moderation (stressful life events) of the relationship between qi stagnation and depression clarifies why and how this relationship occurs.

The results confirmed those of previous studies that the greater the women's qi stagnation constitution was, the higher their depression risk was [10–12]. The findings also supported the constitutional theory of Chinese medicine, which holds that people with the qi stagnation constitution are at increased risk for depression [14]. According to traditional Chinese medicine, individuals with a constitution with an imbalanced state of yin, yang, qi, and blood were more prone to develop some diseases [7, 48–50]. Yin is defined as the material aspects of the organism, and yang refers to the functions [51]. The organs work together by regulating and preserving qi and blood [51]. Qi is the basis for the maintenance of the body's vital activities, and an impeded qi can lead to the loss of mental nourishment as well as emotional frustration [18]. Previous studies examined numerous vulnerabilities and risk factors in association with depression such as genetics, biological functioning, social environments, or personal characteristics in an attempt to understand the origins of this mental disorder [52]. However, with the exception of some Chinese medicine researchers, most scholars have paid little attention to the associated risk factors by adopting a Chinese medicine perspective. This study confirmed that imbalanced constitution of Chinese medicine is indeed a risk factor for the occurrence of depression in a sample of women. Therefore, more attention needs to be paid to the role of constitution in the literature on depression.

Although the relationship between the qi stagnation constitution and depression has been examined, little is known about the mechanism based on empirical evidence. The present study found that rumination mediated the relationship between the qi stagnation constitution and depression. This result confirmed the view that a ruminative response style can be considered as one of the cognitive manifestation of qi stagnation constitution [14] and was consistent with a large body of research showing that rumination is positively associated with depressive symptoms [23, 25]. People with the qi stagnation constitution usually ruminate endlessly [18]. Then, individuals who tend to ruminate are more vulnerable to descend into depression. Rumination makes people more likely to use negative thoughts and memories, interferes with effective problem solving, and reduces social support [25], which may explain the influence of rumination on depression. In addition, rumination partially, rather than fully, mediated the effect of the qi stagnation constitution on depression. That is, the effect through rumination does not entirely account for the association between the qi stagnation constitution and depression. There are likely other factors that explain the variance in the relationship. Additional studies, therefore, should focus on other mechanisms that might explain the vulnerability effect of the qi stagnation constitution on depression. In conclusion, the findings support the view that rumination is a common mechanism between depressive risk factors and subsequent depressive episodes [21].

The results also showed that the relationship between the qi stagnation constitution and depression was markedly stronger for women who reported highly stressful life events compared to those who reported slightly stressful life events. It shows that stressful life events act as a risk-enhancing factor in depression among women with the qi stagnation constitution. One possible explanation is that the liver is thought to be able to regulate qi movement and balance emotion in traditional Chinese medicine; however, the liver qi can easily be affected by stress [53, 54]. Women who reported stagnation of the qi with a weak liver were more likely to have liver dysfunction and were easily overcome by negative mood under stress [18]. These results offer new support for the diathesis-stress model of depression [27].

Additionally, this study further investigated at what stage the stressful life events work in mediating effect. The study found no moderating effect of stressful life events on the relation of qi stagnation constitution and rumination at the first stage. The result supports the view that rumination is a relatively stable trait for qi depression constitution [14], which could not be affected by stressful life events. However, the study found that the moderating effect of stressful life events had function at the second stage of the mediational model—the relationship between rumination and female depression. Compared to women with a low level of stress, women with the qi stagnant constitution who tended to ruminate in response to a high level of stress were more prone to depression. This result, which is consistent with previous results [29–31], suggests that the indirect effect of rumination was significant only in the face of highly stressful life events. That is, stressful life events may serve to strengthen the disadvantage associated with rumination, thus leading the female with qi stagnation constitution to suffering from depression.

However, the sample primarily consisted of female college students in China. The association between the qi stagnation constitution and depression in a diverse sample such as clinically depressed patients should be examined. Additionally, prospective studies with larger samples are needed to test why and how the qi stagnation constitution can affect depression through rumination and stressful life events, and the mechanism between the qi stagnation constitution and depressive symptom maintenance can be further examined using a longitudinal design. Moreover, beyond the qi stagnation constitution, future researchers should also investigate whether lacking vital energy nature, yang deficiency, and other vulnerable constitutions affect depressive symptoms.

In summary, the present data provide empirical evidence of the relationship between the qi stagnation constitution and depression in women using a moderated mediation model. In addition to identifying rumination as a partial mediator of the relationship between the qi stagnation constitution and depression, we also found that stressful life events moderated the direct effect and mediating effect between the qi stagnation constitution and depression. This is of clinical importance, as a vulnerable qi stagnation constitution can be improved and resumed through practices in traditional Chinese medicine. Chinese medicine is the most commonly used complementary or alternative to Western medicine in fully institutionalized parts of Chinese health care [51]. Therefore, additional concern for improving the qi stagnation constitution, reducing rumination, and enhancing coping in the face of stressful life events will help to prevent depression.

## Figures and Tables

**Table 1:** Means, standard deviations, and correlations for the variables.

Predictors	*M*	SD	(1)	(2)	(3)	(4)
(1) Qi stagnation constitution	14.55	4.07	1			
(2) Depression	14.87	7.06	0.48^*∗∗*^	1		
(3) Rumination	42.59	8.69	0.57^*∗∗*^	0.39^*∗∗*^	1	
(4) Stressful life events	31.49	14.88	0.33^*∗∗*^	0.31^*∗∗*^	0.33^*∗∗*^	1

*Note*. ^*∗∗*^*p* < 0.01.

**Table 2:** A mediation model predicting depression.

Predictor	Rumination			Depression		
	*β*	SE	*t*	*β*	SE	*t*
Qi stagnation constitution	1.21	0.05	23.20^*∗∗∗*^	0.66	0.05	12.19^*∗∗∗*^
Rumination				0.14	0.03	5.64^*∗∗∗*^

Indirect effect						
Effect	SE			LLCI		ULCI

0.17	0.04			0.10		0.25

*Note.* SE = standard error. LLCI/ULCI = lower limit confidence interval/upper limit confidence interval. Reported coefficients are unstandardized ordinary least squares regression coefficients; ^*∗∗∗*^*p* < 0.001.

**Table 3:** A moderated mediation model predicting depression.

Predictor	Rumination			Depression		
	*β*	SE	*T*	*β*	SE	*T*
Qi stagnation constitution	1.10	0.05	23.20^*∗∗∗*^	0.57	0.05	10.52^*∗∗∗*^
Stress				0.06	0.01	4.38^*∗∗*^
Rumination				0.14	0.03	4.40^*∗∗∗*^
Rumination × stress				0.01	0.002	3.04^*∗∗*^
Qi stagnation constitution × stress				0.01	0.003	3.13^*∗∗*^

	Conditional direct effect and indirect effect					
		Effect	SE	LLCI	ULCI	

Conditional direct effect						
Low stress		0.41	0.08	0.25	0.56	
Medium stress		0.57	0.05	0.46	0.67	
High stress		0.72	0.07	0.59	0.85	
Conditional indirect effect						
Low stress		0.06	0.04	−0.02	0.14	
Medium stress		0.14	0.04	0.08	0.21	
High stress		0.23	0.06	0.12	0.35	

*Note*. Stress = stressful life events; *p* value: analysis by ordinary least squares regression through PROCESS macro. ^*∗*^Significant at *p* < 0.05; ^*∗∗*^significant at *p* < 0.01; ^*∗∗∗*^significant at *p* < 0.001.
